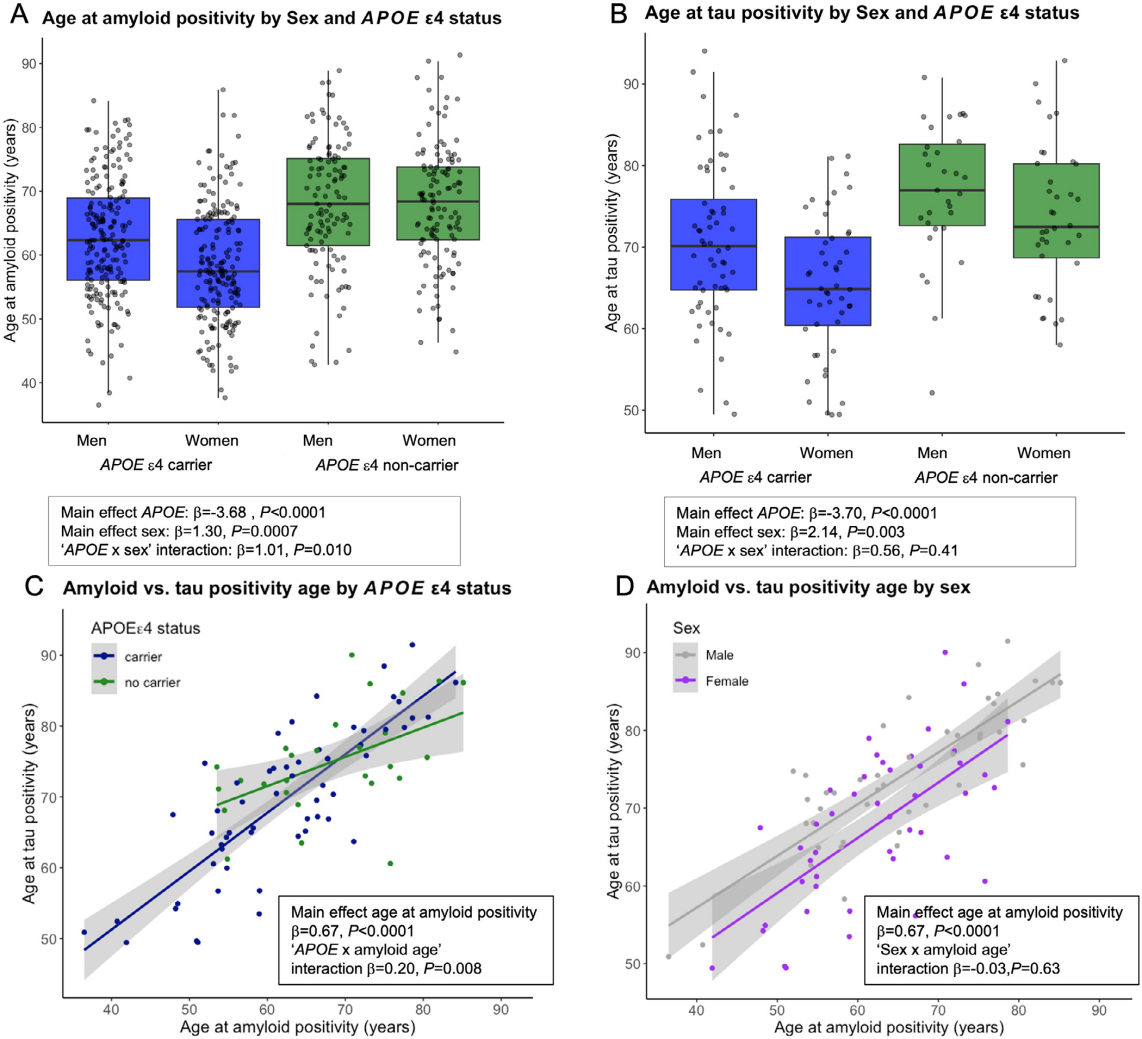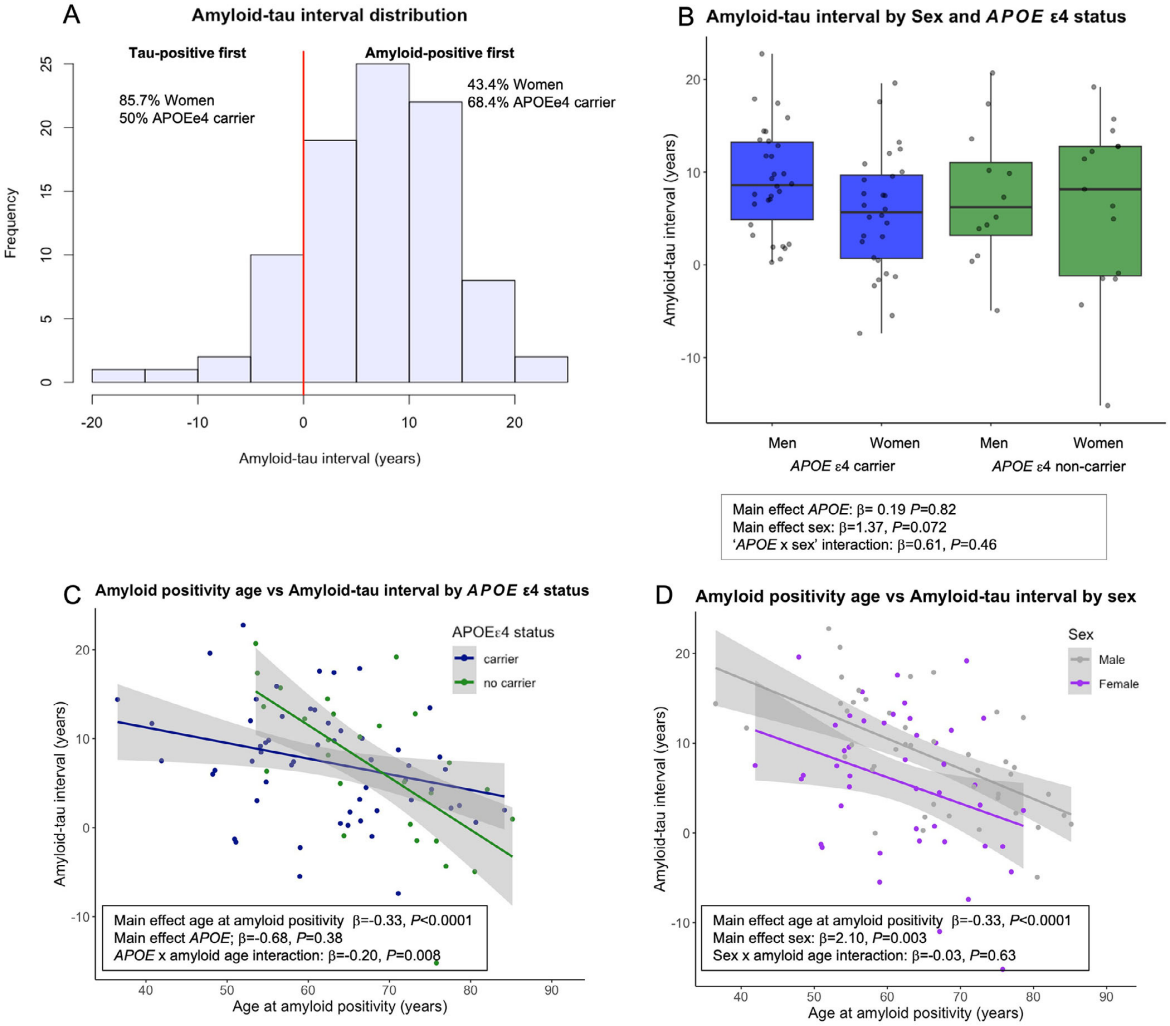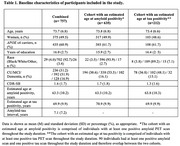# Effects of APOE ε4 and sex on estimated ages at amyloid and tau PET positivity

**DOI:** 10.1002/alz70860_104392

**Published:** 2025-12-23

**Authors:** Marta Milà‐Alomà, Zachary Hausle, Kellen K. Petersen, Pamela Zobel‐Thropp, Suzanne E. Schindler, Duygu Tosun

**Affiliations:** ^1^ Department of Radiology and Biomedical Imaging, University of California, San Francisco, San Francisco, CA, USA; ^2^ Department of Neurology, Washington University School of Medicine, St. Louis, MO, USA

## Abstract

**Background:**

AD is characterized by the sequential accumulation of amyloid and tau, but the interplay between these pathologies and other factors remains unclear. Using amyloid and tau PET clocks, we examined the temporal relationships between amyloid and tau positivity and potential modifying effects of sex and *APOE*ε4.

**Method:**

We studied 757 ADNI participants with longitudinal amyloid (18F‐Florbetapir) or tau (18F‐Flortaucipir) PET imaging. Of these, 635 had at least one positive amyloid PET scan (SUVR>0.78), and 212 had at least one positive tau PET scan (mesial‐temporal SUVR>1.41), enabling the estimation of age at amyloid or tau positivity (Table 1). Linear regression models assessed the effects of sex and *APOE*ε4 on the ages at amyloid and tau positivity. Ninety individuals had estimates of the age at positivity for both amyloid and tau, allowing estimation of the amyloid‐tau time interval. Regression analyses examined the association between amyloid and tau positivity ages. Additional models included interaction terms to explore potential modifying effects of sex and *APOE*ε4.

**Result:**

Men and *APOE*ε4 non‐carriers had an older age at amyloid and tau positivity. For amyloid positivity age, there was a significant interaction between sex and *APOE*ε4 status, with sex modifying amyloid positivity age in *APOE*ε4 carriers. (Figure 1A, B). *APOE*ε4 status, but not sex, modified the association between age at amyloid and at tau positivity (Figure 1C, D). Most individuals (84.5%) became amyloid positive before tau positive and those who became tau positive first were more likely to be women (Figure 2A, B). The amyloid‐tau interval was shorter in individuals with an older age at amyloid positivity (Figure 2C, D), and this association was more pronounced in *APOE*ε4 non‐carriers (Figure 2C). Men had a longer amyloid‐tau interval than women, regardless of the age at amyloid positivity (Figure 2D).

**Conclusion:**

Our findings suggest that a later age at amyloid positivity is associated with earlier tau positivity and this association differs by *APOE*ε4 carriership status and sex. These results may imply differences in tau‐related disease progression and highlight the need to consider sex and *APOE*ε4 status in clinical trials aimed at slowing the onset of tau pathology.